# A model-guided alternating high-temperature thermotherapy achieves complete eradication of sugarcane mosaic virus and preserves physiological integrity in *Saccharum officinarum* cv. Xuezhe

**DOI:** 10.3389/fpls.2026.1752582

**Published:** 2026-02-19

**Authors:** Guo-Qiang Huang, Feng Li, Jian-Xia Zhong, Jian-Peng Zhang, Jin-Long Guo, Qian Dong, Tung-Yu Hsieh

**Affiliations:** 1 National Engineering Research Center of Sugarcane, Fujian Agriculture and Forestry University, Fuzhou, China; 2 Wuyou Ecological Agriculture Co., Ltd., Fuqing, China; 3 Fujian Tianyuan Shiguang Leisure Agriculture Co., Ltd., Fuqing, China; 4 Lanxi Agricultural Technology Co., Ltd., Fuqing, China; 5 Cai Die Biological Technology Co., Ltd., Yongtai, China; 6 Fujian Universities and Colleges Engineering Research Center of Modern Facility Agriculture, Fujian Polytechnic Normal University, Fuqing, China; 7 College of Food and Biological Engineering, Fujian Polytechnic Normal University, Fuqing, China; 8 Key Laboratory of Genetics, Breeding and Multiple Utilization of Crops, Ministry of Education, Fujian Agriculture and Forestry University, Fuzhou, China; 9 Key Laboratory of Biological Breeding for Fujian and Taiwan Crops, Ministry of Agriculture and Rural Affairs, Fujian Agriculture and Forestry University, Fuzhou, China

**Keywords:** axillary-bud cuttings (ABCs), catalase (CAT), classifier modeling, peroxidase (POD), redox homeostasis, response surface optimization, uniform-design experiment

## Abstract

**Introduction:**

Sugarcane mosaic virus (SCMV) undermines both yield and eating quality of the chewing-cane cultivar *Saccharum officinarum* cv. Xuezhe. Although constant high-temperature thermotherapy can eliminate SCMV, it frequently causes serious heat injury in heat-sensitive genotypes. This study developed an alternating high-temperature thermotherapy (AHTT) scheme to eradicate SCMV while maintaining axillary-bud vigor on rooted cuttings.

**Methods:**

A uniform-design experiment combined with quadratic regression analysis was used to optimize three factors: maximum treatment temperature (42–48 °C), duration of the high-temperature pulse (2–6 h in darkness), and the subsequent recovery phase at 38 °C (3–6 h under light). These cycles were applied daily over a 12-day regimen. Outcomes included sprouting, rooted-cutting performance, and virus elimination.

**Results:**

The model predicted an optimal schedule of 47 °C for 5 h (dark) followed by a 7-h recovery at 38 °C, repeated daily for 12 days; this schedule was experimentally validated. Under the optimized regime, axillary buds on 12-day-rooted cuttings were completely SCMV-free by RT-PCR, with 97% sprouting and acceptable vigor. AHTT was also associated with increased catalase (CAT) activity and reduced peroxidase (POD) activity.

**Discussion:**

The model-guided AHTT protocol reconciles effective SCMV eradication with acceptable plant stress and performs better than constant-temperature thermotherapy. It provides a practical, scalable, and environmentally friendly approach to generate virus-free sugarcane planting material, with potential relevance to other vegetatively propagated, heat-sensitive crops.

## 1 Introduction

Sugarcane mosaic disease, caused predominantly by Sugarcane mosaic virus (SCMV) and often accompanied by co-infections with Sorghum mosaic virus (SrMV) and Sugarcane streak mosaic virus (SCSMV), remains a major constraint on sugarcane cultivation worldwide (Lu et al., 2021; Daugrois et al., 2024). Infected plants typically show reduced sprouting, weakened photosynthetic capacity, shortened internodes, and fewer tillers, all of which ultimately compromise sugar accumulation and yield. These impacts have been well documented across production regions, with yield losses in severe cases exceeding 50% (Lu et al., 2021; Srivastava et al., 2024). The chewing-cane cultivar *Saccharum officinarum* cv. Xuezhe—valued for its thick stalks and desirable fresh-eating quality—has proven particularly vulnerable, and under conventional vegetative propagation systems, virus incidence in farmers’ fields can approach complete infection. This situation highlights an urgent need for reliable sanitation strategies capable of producing healthy planting material and preserving the performance of this commercially important cultivar.

Sanitizing planting material remains a cornerstone of virus-management strategies and is especially critical for germplasm conservation and the production of virus-free mother plants (Wang et al., 2018). Among the sanitation approaches, thermotherapy—implemented either as *in vitro* hot-air or hot-water exposure—has proven to be cost-effective and broadly applicable (Wang et al., 2018; Szabó et al., 2024). Its mode of action is assumed to encompass inhibition of viral replication, destabilization of viral movement proteins, and enhanced host antiviral RNA-silencing responses under elevated temperatures (Boyko et al., 2000; Tsai et al., 2022; Atabekova et al., 2024). However, standard constant-temperature thermotherapy regimes (e.g., ≥ 52 °C for sugarcane axillary buds) often impose unacceptable lethality or severely retard bud outgrowth in heat-sensitive genotypes (Benda, 1972; Wagih et al., 2009), rendering them less feasible for practical application in cultivars like Xuezhe.

Recent reviews emphasize a shift toward more refined eradication protocols, including alternating temperature schedules (Wang et al., 2018), integration with meristem-tip culture (Vivek and Modgil, 2018), chemotherapy or cryotherapy (Wang et al., 2009, 2018; Bettoni et al., 2022a, 2024; Sarropoulou et al., 2024), and quantitative modeling of treatment parameters. For example, a 2024 mini-review on *in vitro* thermotherapy across woody plants highlighted improved outcomes when alternating high/low temperature cycles were used as opposed to static treatments (Szabó et al., 2024). Meanwhile, a 2024 study in *Actinidia macrosperma* infected with Actinidia virus A (AcVA), Actinidia virus B (AcVB), and Actinidia chlorotic ringspot-associated virus (AcCRaV) reported that combining thermotherapy and shoot tip cryotherapy yielded significantly higher virus-free recovery rates (Zhang et al., 2024). This synergistic effect is often attributed to heat-induced suppression of viral replication and reduced viral titre, together with the preferential survival/regeneration of minimally infected meristematic cells after cryotherapy, thereby enhancing shoot-tip virus eradication. Despite these advances, few studies have systematically optimized alternating high-temperature thermotherapy (AHTT) for SCMV elimination in chewing cane, especially at the axillary-bud stage and under hot-air (rather than hot-water) regimes (Cheong et al., 2012; Cele et al., 2024).

Here, we introduce a hot-air Alternating High-Temperature Thermotherapy (AHTT) protocol designed specifically for SCMV-infected Xuezhe axillary-buds from rooted cuttings. Using a uniform-design experimental framework (Fang and Lin, 2003), we dissect three controllable factors—upper-pulse temperature (42–48 °C), high-temperature pulse duration (2–6 h, dark) and recovery duration at 38 °C (3–6 h, light)—to (i) evaluate rooted cutting emergence and vigor, (ii) monitor leaf physiological responses (nitrogen balance index, chlorophyll surrogates (Cerovic et al., 2012; Dong et al., 2021), antioxidant enzyme activities (Mishra et al., 2023; Mahajan et al., 2025; Samat et al., 2025), and (iii) determine true SCMV elimination via RT-PCR (Xie et al., 2009). We further calibrate a quadratic regression model (Heckert et al., 2002; Hsieh et al., 2011) to predict elimination rates and validate the optimal regime experimentally. This work aims not only to deliver a practical sanitation protocol for chewing-cane clean-stock production, but also to provide a modeling and process-design framework that could be generalized to other heat-sensitive crops in need of virus-free propagation (Mishra et al., 2023; Hsieh et al., 2025).

## 2 Materials and methods

### 2.1 Plant materials and verification of infection

SCMV-infected stalks of *S. officinarum* cv. Xuezhe were collected from the Jinshan Experimental Station of the National Engineering Research Center of Sugarcane (Fujian Agriculture and Forestry University, Fuzhou, China). Source plants were first screened by one-step RT-PCR to confirm SCMV infection (Xie et al., 2009) and only RT-PCR-positive plants were used as donor material. In April 2022, symptomatic stalks were cut into single-node setts, each containing one axillary bud (single-bud cuttings), which served as the starting material for thermotherapy and subsequent regeneration. Single-bud cuttings (hereafter referred to as axillary-bud cuttings, ABCs) were prepared from SCMV-infected source plants. After 12 consecutive days of AHTT, axillary buds developed into axillary-bud shoots (ABSs) bearing 3–4 true leaves, and SCMV diagnosis (one-step RT-PCR) was performed at this time point using sampled leaves.

### 2.2 Chemicals and reagents

Analytical-grade reagents (≥ 99.5% purity) were obtained from Sinopharm Chemical Reagent Co., Ltd. Guaiacol and other substrates used for enzymatic assays were of AR grade. The TransZol Up Plus RNA Kit and TransScript One-Step RT-PCR SuperMix Kit were purchased from TransGen Biotech (Beijing, China).

### 2.3 Instruments and growth conditions

AHTT treatments were conducted using a programmable artificial-climate chamber (Model RXZ-160A; Zhongxin Medical Instrument Co., Jiaxing, China). Photosynthetic photon flux density (PPFD) was maintained at ≈ 120 µmol m^-^² s^-^¹ during light cycles. PCR amplification was performed using a PCR thermal cycler (Hangzhou Bioer Technology, Hangzhou, China). RNA quantification employed a NanoDrop One spectrophotometer (Thermo Fisher Scientific, USA). Physiological parameters were measured using a Dualex 4 Scientific Leaf Clip (Force-A, France), which enables simultaneous non-destructive measurement of chlorophyll, epidermal flavonol, and nitrogen balance index (Cerovic et al., 2012; Dong et al., 2021).

### 2.4 Establishment of single-bud cultures and alternating high-temperature thermotherapy

**Figure 1:**
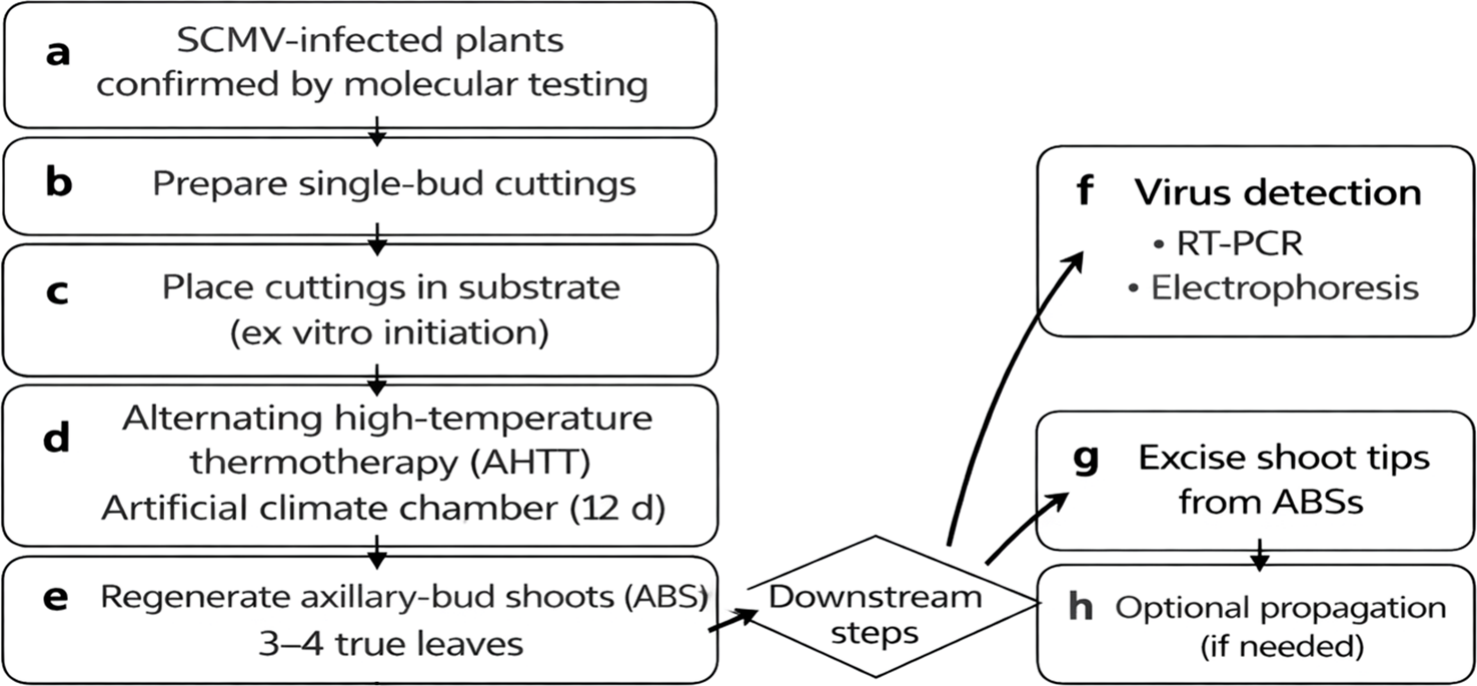
Experimental workflow for model-guided alternating high-temperature thermotherapy (AHTT) and subsequent SCMV detection/tissue culture. **(a)** Single-bud cuttings (SCMV infection confirmed by molecular testing); **(b)** cuttings were placed in the culture medium/substrate; **(c)** cuttings were treated in an artificial climate chamber under AHTT for 12 days; **(d)** cuttings developed into axillary-bud shoots/cuttings (ABSs/ABCs) bearing 3–4 true leaves; **(e)** total RNA was extracted from leaves for RT-PCR; **(f)** virus detection by RT-PCR and electrophoresis; **(g)** excision of ABC shoot tips; **(h)** tissue culture for regeneration.

Sugarcane was propagated using single-node stem cuttings (single-node setts; single-bud setts) excised from the middle portion of physiologically mature stalks (the topmost and basal portions were avoided to minimize variation in bud maturity). Each sett was 10-15 cm in length and 3-4 cm in diameter, and contained one node bearing a single axillary bud. Stalks were cut at the midpoints of adjacent internodes, so that the node was positioned approximately in the middle of the sett. Single-node cuttings were rinsed thoroughly under running water, surface-sterilized in 0.05% (v/v) carbendazim for 15 min, and washed three times with sterile distilled water. Single-node cuttings (hereafter referred to as axillary-bud cuttings, ABCs) were then embedded horizontally in vessels containing an approximately 8-cm-thick 2:1:1 (v/v/v) peat soil:perlite:vermiculite substrate, with ~2 cm of the same substrate gently covering the setts above the buds, and preconditioned at 38 °C in darkness for 24 h. Thereafter, ABCs were subjected to alternating high-temperature thermotherapy (AHTT) for 12 consecutive days. Each daily cycle consisted of (i) a high-temperature pulse (X_1_, 42-48 °C) applied for X_2_ h in darkness, immediately followed by (ii) a recovery phase at 38 °C applied for X_3_ h under light. Outside the AHTT window, cultures were maintained at 28 ± 1 °C to complete a 24 h cycle under the same photoperiod. The overall experimental workflow is summarized in **Figure 1**.

Six AHTT regimes were generated using a U6 (3^2^ x 2^1^) uniform-design matrix (**Table 1**). The three controllable factors were (X_1_) high-temperature level (42-48 °C), (X_2_) duration of the high-temperature pulse (2–6 h, dark), and (X_3_) duration of the 38 °C recovery phase (3–6 h, light). For each regime, 10 ABCs were treated per replicate, with three biological replicates. Here, one biological replicate corresponded to an independent batch of 10 ABCs (10 single-node setts) processed and cultured in parallel; thus, each AHTT regime included 3 biological replicates (n = 30 ABCs in total). A non-thermotherapy control was maintained at 28 ± 1 °C under a 12 h photoperiod. After 12 days, axillary buds developed into axillary-bud shoots (ABSs) bearing 3–4 true leaves. Leaves were sampled for RT-PCR detection of SCMV. For optional downstream multiplication, shoot tips from RT-PCR-negative ABSs may be excised and used for tissue-culture propagation (see **Figure 1**).

**Table 1:** Uniform design scheme of U6 (3^2^×2^1^).

Group	Factor X_1_ high-temperature/°C	Factor X_2_ time for high-temperature/h in dark	Factor X_3_ time for 38 °C/h in light (120 µmol m^-^² s^-^¹)
D1-42-2-6	42	2	6
D4-42-4-3	42	4	3
D5-45-6-6	45	6	6
D2-45-2-3	45	2	3
D6-48-4-6	48	4	6
D3-48-6-3	48	6	3

### 2.5 Growth measurements

**Figure 2:**
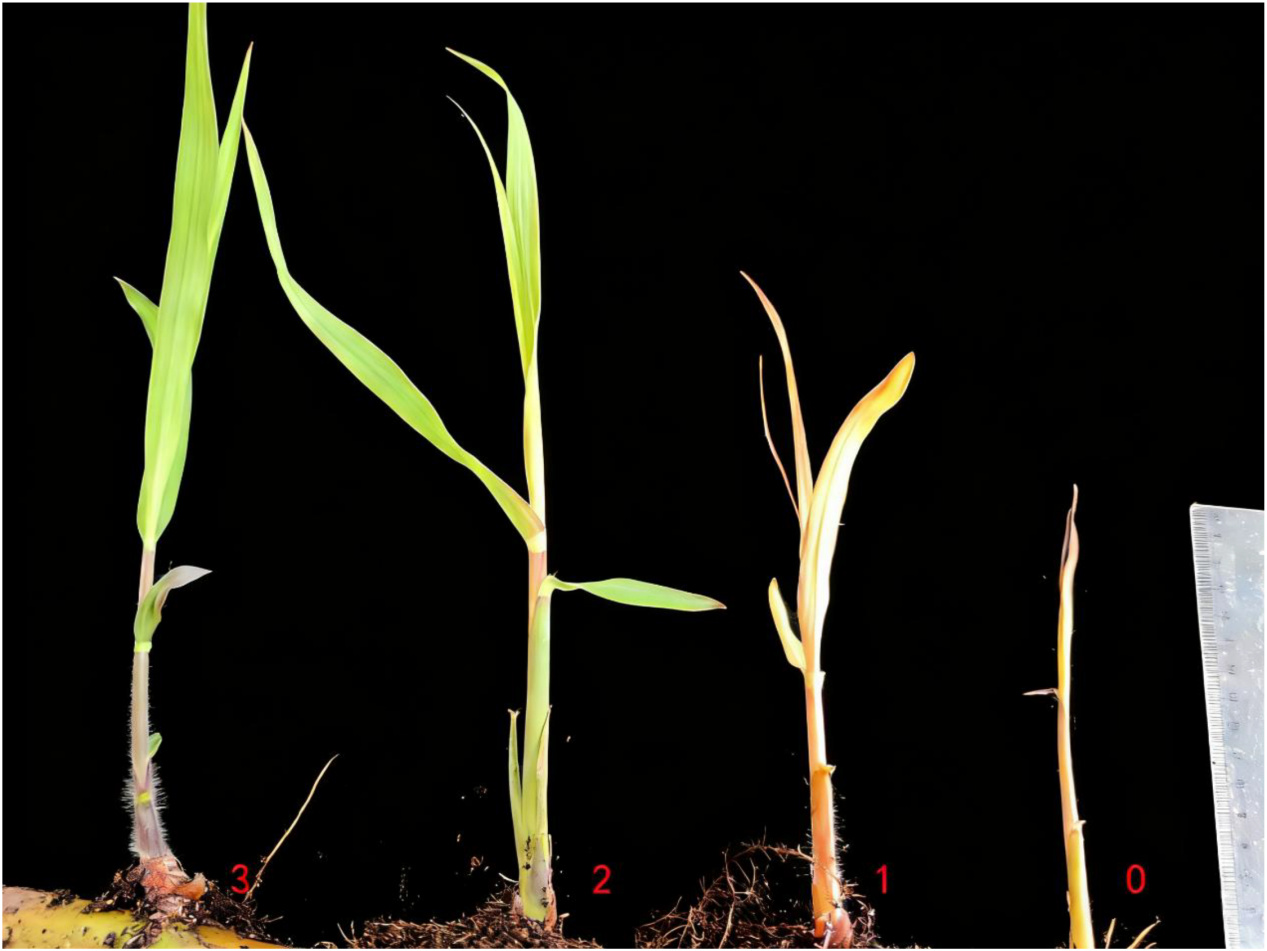
Representative photographs illustrating the visual health index (0–3) used to score axillary-bud shoots. Scores correspond to: 3 = very healthy with vigorous green leaves; 2 = healthy with mainly green leaves and partial yellowing; 1 = slightly healthy with mainly yellow leaves and some green tissue; 0 = poor health with yellow buds without expanded leaves.

Sprouting percentage (%) was calculated as (number of sprouted buds > 5 mm tall/total number of buds) × 100 (see **Figure 2** for representative images used to score the health index). Axillary-bud shoot (ABS) cutting height was measured from the soil surface to the apical tip using a ruler with 0.1 cm precision. Health indices (0–3 scale) were visually scored based on leaf/bud appearance (**Figure 2**) as follows: 3 = very healthy, vigorous green leaves; 2 = healthy, mainly green leaves with partial yellowing; 1 = slightly healthy, mainly yellow leaves with some green tissue; 0 = poor health, yellow buds without expanded leaves.

### 2.6 Leaf physiological indices and antioxidant enzyme activities

Physiological indices were measured on the +1 functional leaf (the top visible dewlap leaf). The nitrogen balance index (NBI), chlorophyll (Chl), and epidermal flavonol (Flav) contents were recorded using the Dualex sensor (Cerovic et al., 2012; Dong et al., 2021). Peroxidase (POD; EC 1.11.1.7) and catalase (CAT; EC 1.11.1.6) activities were determined following a modified spectrophotometric protocol (Chance and Maehly, 1955; Aebi, 1984; Gao, 2006) with minor adjustments for micro-volume assays. One unit of POD activity corresponded to a ΔA_470_ of 0.01 min^-^¹ g^-^¹ FW; one unit of CAT activity to a ΔA_240_ of 0.1 min^-^¹ g^-^¹ FW. Each assay included three technical replicates.

### 2.7 Detection of SCMV by one-step RT-PCR

Total RNA was extracted from +1 functional leaves using the TransZol Up Plus Kit. RNA integrity and purity were verified by NanoDrop spectrophotometry (A_260_/A_280_ ≈ 2.0). SCMV detection was conducted via one-step RT-PCR (TransScript SuperMix Kit) following Xie et al. (2009) with the degenerate primers: F 5′-GAAGAXGTYTTCCAYCAAFCXGGAAC-3′ and R 5′-AGCTGTGTGTCTCTCTGTATTCTC-3′. Amplifications (20 µL total volume) contained 2× reaction mix (10 µL), RNA template (2 µL), primer pair (0.4 µL each), enzyme mix (0.4 µL), and RNase-free water (6.8 µL). Cycling conditions: 45 °C 30 min (reverse transcription); 94 °C 3 min pre-denaturation; 35 cycles of 94 °C 30 s, 55 °C 30 s, 72 °C 1 min; final extension 72 °C 10 min. PCR products (906 bp) were resolved on 1% agarose gels and visualized under UV. Virus-elimination rates were computed from RT-PCR results across three replicates. The detection workflow aligns with recent updates to sugarcane virus diagnostics in multiplex or one-step systems (Srivastava et al., 2024).

### 2.8 Model-based optimization and verification

Elimination rates were calculated from RT-PCR results across the three biological replicates for each AHTT regime. A quadratic regression model based on the uniform-design matrix was fitted to relate SCMV elimination rate to X_1_, X_2_, and X_3_, and response-surface optimization was used to identify an operational regime that maximized virus elimination while maintaining acceptable shoot vigor. The model-derived optimal AHTT schedule (47 °C for 5 h in darkness followed by 38 °C for 7 h under light, repeated daily for 12 days) was then validated using three independent replicates of 10 ABCs each. After treatment, leaves at positions +1 to +3 were tested by RT-PCR to confirm virus eradication and assess the physiological quality of the resulting ABSs.

### 2.9 Statistical analysis

One-way ANOVA followed by Duncan’s multiple-range test (p < 0.05) was performed for growth and physiological indices using IBM SPSS v22 (IBM Corp, 2013). Regression analysis and response-surface modeling for classifier-based (Hsieh et al., 2011; Chiou et al., 2015) elimination rate were performed in Minitab v19 (Minitab, 2019) according to the uniform design matrix. Model performance was assessed using R², adjusted R², predicted R², and p-values.

## 3 Results

### 3.1 Rooted cutting emergence and vigor under AHTT

**Figure 3:**
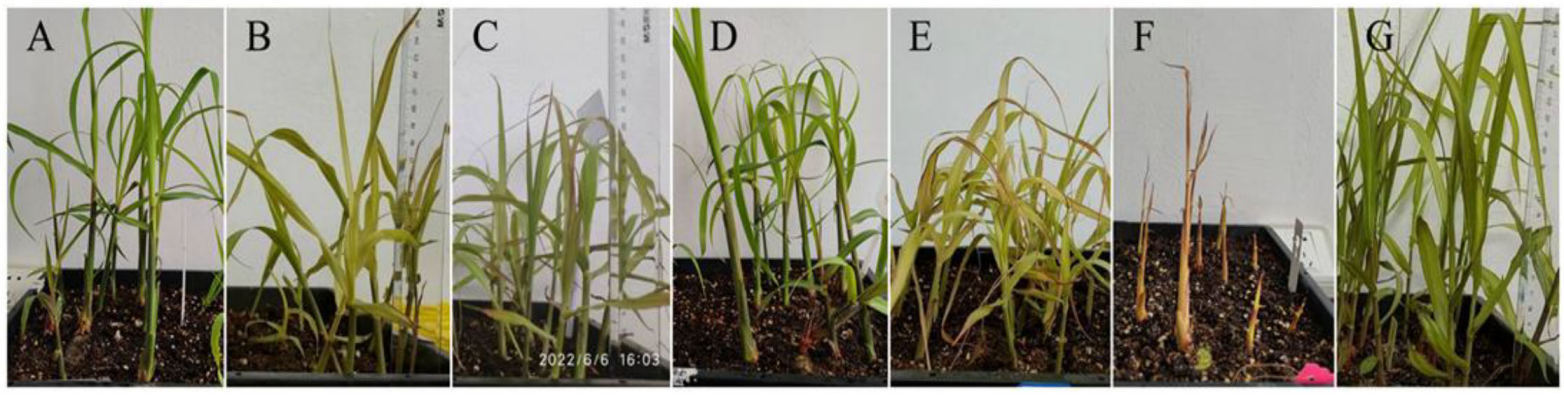
Growth of ABSs under different AHTT regimen. **(A)** D1-42-2-6, represented the AHTT treatment with the temperatures alternating between 42 °C 2 h dark and 38 °C 6 h light (AHTT, 42 °C 2 h/38 °C 6 h); **(B)** D4-42-4-3, represented AHTT, 42 °C 4 h/38 °C 3 h; **(C)** D5-45-6-6, represented AHTT, 45 °C 6 h/38 °C 6 h; **(D)** D2-45-2-3, represented AHTT, 45 °C 2 h/38 °C 3 h; **(E)** D6-48-4-6, represented AHTT, 48 °C 4 h/38 °C 6 h; **(F)** D3-48-6-3, represented AHTT, 48 °C 6 h/38 °C 3 h; **(G)** CK, represented the control treatment with normal temperature (28 ± 1 °C).

Across all alternating high-temperature thermotherapy (AHTT) regimens, sprouting percentages were comparable to the control and showed no significant reduction (**Table 2**). In contrast, axillary-bud shoot height and health index were treatment-sensitive. Short high-temperature pulses (≤ 2 h) supported better growth: groups D1-42-2–6 and D2-45-2–3 produced axillary-bud shoot significantly taller than the control and all other treatments (p < 0.01), with health index = 3 and uniformly green leaves. Longer or more severe exposures depressed vigor: D3-48-6–3 exhibited the lowest health index (0) and severe growth suppression, and D6-48-4–6 also showed reduced height and chlorosis (**Figure 3**). Within the tested window, recovery at 38 °C did not measurably improve height or health beyond the effect of shortening the high-temperature pulse, indicating that pulse severity dominated growth outcomes.

**Table 2:** Effects of different AHTT treatments on the growth index of ABCs.

Group	Sprouting percentage (%)	Axillary-bud shoot height	Health indices
CK	83.3 ± 15.3 ^a^	7.70 ± 0.67 ^Bb^	3
D1-42-2-6	100.0 ± 0.0 ^a^	12.32 ± 3.14 ^Aa^	3
D4-42-4-3	100.0 ± 10.0 ^a^	4.12 ± 0.30 ^Bc^	2
D5-45-6-6	90.0 ± 10.0 ^a^	6.58 ± 1.12 ^Bbc^	2
D2-45-2-3	100.0 ± 0.0 ^a^	13.03 ± 1.74 ^Aa^	3
D6-48-4-6	93.3 ± 5.8 ^a^	4.58 ± 1.18 ^Bc^	1
D3-48-6-3	91.7 ± 14.4 ^a^	4.53 ± 0.44 ^Bc^	0

^1^mean ± S.D.

^2^lowercase letters indicate significant difference at *p* < 0.05; capital letters indicate significant difference at *p* < 0.01.

^3^Health index scores were defined as follows: 3, very healthy (green leaves); 2, healthy (mainly green with partial yellowing); 1, slightly healthy (mainly yellow with some green tissue); 0, poor health (yellow buds without expanded leaves).

### 3.2 Leaf physiological responses

Dualex readings revealed clear temperature–time dependencies. Relative to the control, chlorophyll (Chl) and nitrogen balance index (NBI) decreased under AHTT, but remained higher when the high-temperature pulse was 2 h (D1-42-2-6, D2-45-2-3). Extending the pulse to 4–6 h caused a marked decline in Chl and NBI (p < 0.01), consistent with aggravated heat stress and transient nitrogen-metabolism suppression (**Table 3**). Antioxidant enzymes showed divergent trends: POD activity decreased progressively with increasing thermal severity (as low as 16.00 U g^-^¹ FW min^-^¹ in D6-48-4-6), whereas CAT activity increased from ~6–8 U g^-^¹ FW min^-^¹ (milder regimens) to ~16 U g^-^¹ FW min^-^¹ (D6-48-4-6), indicating a shift in ROS-scavenging strategy under stronger heat load (**Table 4**).

**Table 3:** Effects of different AHTT treatments on the nitrogen balance index (NBI).

Group	NBI	Flav	Anth	Chl
CK	13.426 ± 0.861 ^Aa^	0.980 ± 0.046 ^Aab^	0.232 ± 0.016 ^De^	13.105 ± 0.221 ^Aa^
D1-42-2-6	10.173 ± 1.014 ^Bb^	1.061 ± 0.091 ^Aab^	0.300 ± 0.014 ^Bc^	10.607 ± 0.370 ^Bb^
D4-42-4-3	4.419 ± 0.459 ^Dd^	1.119 ± 0.103 ^Aa^	0.344 ± 0.004 ^Ab^	4.638 ± 0.102 ^Dd^
D5-45-6-6	7.176 ± 0.157 ^Cc^	0.922 ± 0.073 ^Ab^	0.341 ± 0.003 ^Ab^	6.384 ± 0.256 ^Cc^
D2-45-2-3	12.811 ± 0.922 ^Aa^	1.055 ± 0.127 ^Aab^	0.267 ± 0.013 ^Cd^	13.207 ± 1.360 ^Aa^
D6-48-4-6	4.962 ± 0.398 ^Dd^	0.914 ± 0.074 ^Ab^	0.371 ± 0.014 ^Aa^	3.907 ± 0.176 ^Dd^
D3-48-6-3	/	/	/	/

^1^mean ± S.D.

^2^lowercase letters indicate significant difference at *p* < 0.05; capital letters indicate significant difference at *p* < 0.01.

^3^“/” indicates that no functional leaves developed; therefore, measurements were not available.

**Table 4:** Effects of different AHTT treatments on peroxidase (POD) and catalase activity (CAT).

Group	POD activity U (g FW)^-^¹ min^-^¹	CAT activity U (g FW)^-^¹ min^-^¹
CK	3064.00 ± 194.36 ^Aa^	6.10 ± 0.52 ^Cc^
D1-42-2-6	799.56 ± 77.75 ^Cc^	7.50 ± 0.80 ^Cc^
D4-42-4-3	356.00 ± 28.00 ^Dd^	7.77 ± 0.92 ^Cc^
D5-45-6-6	274.67 ± 25.72 ^Dd^	12.50 ± 0.80 ^Bb^
D2-45-2-3	1491.11 ± 120.74 ^Bb^	11.67 ± 0.85 ^Bb^
D6-48-4-6	16.00 ± 6.93 ^Ee^	15.83 ± 1.65 ^Aa^
D3-48-6-3	/	/

^1^mean ± S.D.

^2^lowercase letters indicate significant difference at *p* < 0.05; capital letters indicate significant difference at *p* < 0.01.

^3^“/” indicates that no functional leaves developed; therefore, measurements were not available.

### 3.3 SCMV elimination across AHTT regimens

**Figure 4:**
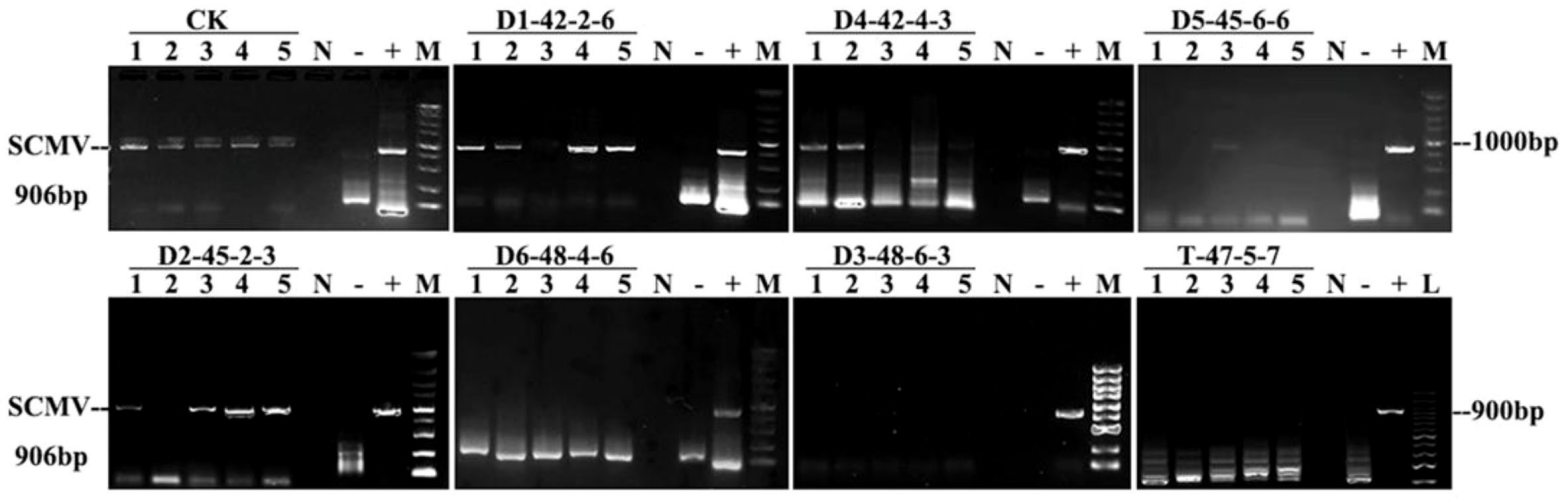
Electrophoretic detection of SCMV RT-PCR amplification products on the 1.0% agarose gels. Lanes L, 100 bp DNA marker; M, 5000 bp DNA marker; +, positive control (SCMV); -, negative control; N, no template control; 1-5, samples of different AHTT treatments. The SCMV positive PCR product size is 906 bp.

RT-PCR (906 bp amplicon) confirmed 100% infection in the control and regimen-dependent elimination among AHTT treatments (**Figure 4**). No elimination occurred at 42 °C/2 h → 38 °C/6 h (0%), whereas 42 °C/4 h → 38 °C/3 h yielded ~55% elimination. Increasing severity improved eradication: 45 °C/6 h → 38 °C/6 h reached ~78%, while 48 °C/4 h → 38 °C/6 h and 48 °C/6 h → 38 °C/3 h both achieved 100% elimination (**Table 5**).

**Table 5:** Effect of the AHTT on the SCMV elimination rate.

No.	X_1_ (High-temperature/°C)	X_2_ (X_1_ processing time/h)	X_3_ (Processing time at 38 °C/h)	Elimination rate/%	Average of elimination rate/%
1	42	2	6	0.0	
2	42	2	6	0.0	0.0 ± 0.0 ^Ee^
3	42	2	6	0.0	
4	42	4	3	55.6	
5	42	4	3	50.0	55.2 ± 5.0 ^Cc^
6	42	4	3	60.0	
7	45	6	6	75.0	
8	45	6	6	77.8	77.6 ± 2.5 ^Bb^
9	45	6	6	80.0	
10	45	2	3	10.0	
11	45	2	3	10.0	10.4 ± 0.6 ^Dd^
12	45	2	3	11.1	
13	48	4	6	100.0	
14	48	4	6	100.0	100.0 ± 0.0 ^Aa^
15	48	4	6	100.0	
16	48	6	3	100.0	
17	48	6	3	100.0	100.0 ± 0.0 ^Aa^
18	48	6	3	100.0	
CK1	28 ± 1	/	/	0.0	
CK2	28 ± 1	/	/	0.0	0.0 ± 0.0 ^Ee^
CK3	28 ± 1	/	/	0.0	

^1^Average of elimination rate was shown as the mean ± S.D.

^2^lowercase letters indicate significant difference at *p* < 0.05; capital letters indicate significant difference at *p* < 0.01.

^3^“/” indicates that no functional leaves developed; therefore, measurements were not available.

### 3.4 Model inference from the uniform-design experiment

**Figure 5:**
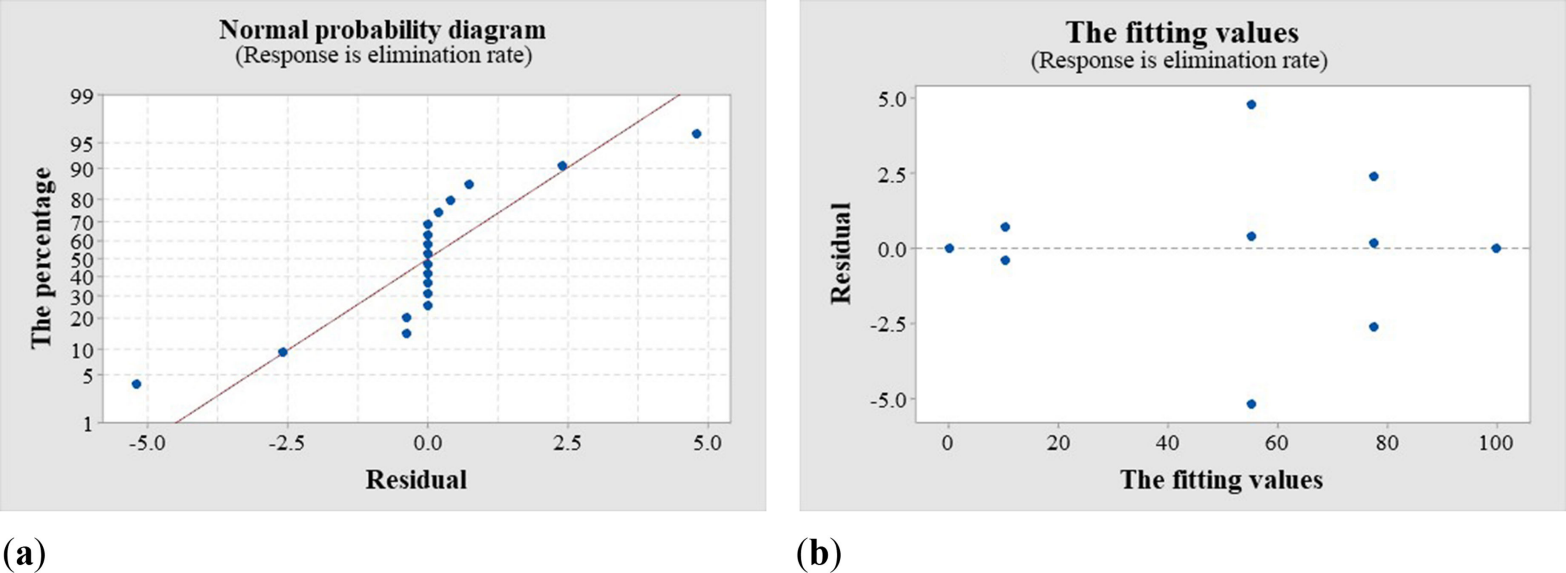
Analysis graph. **(a)** Normal probability of SCMV elimination; **(b)** Residual probability of SCMV elimination.

**Figure 6:**
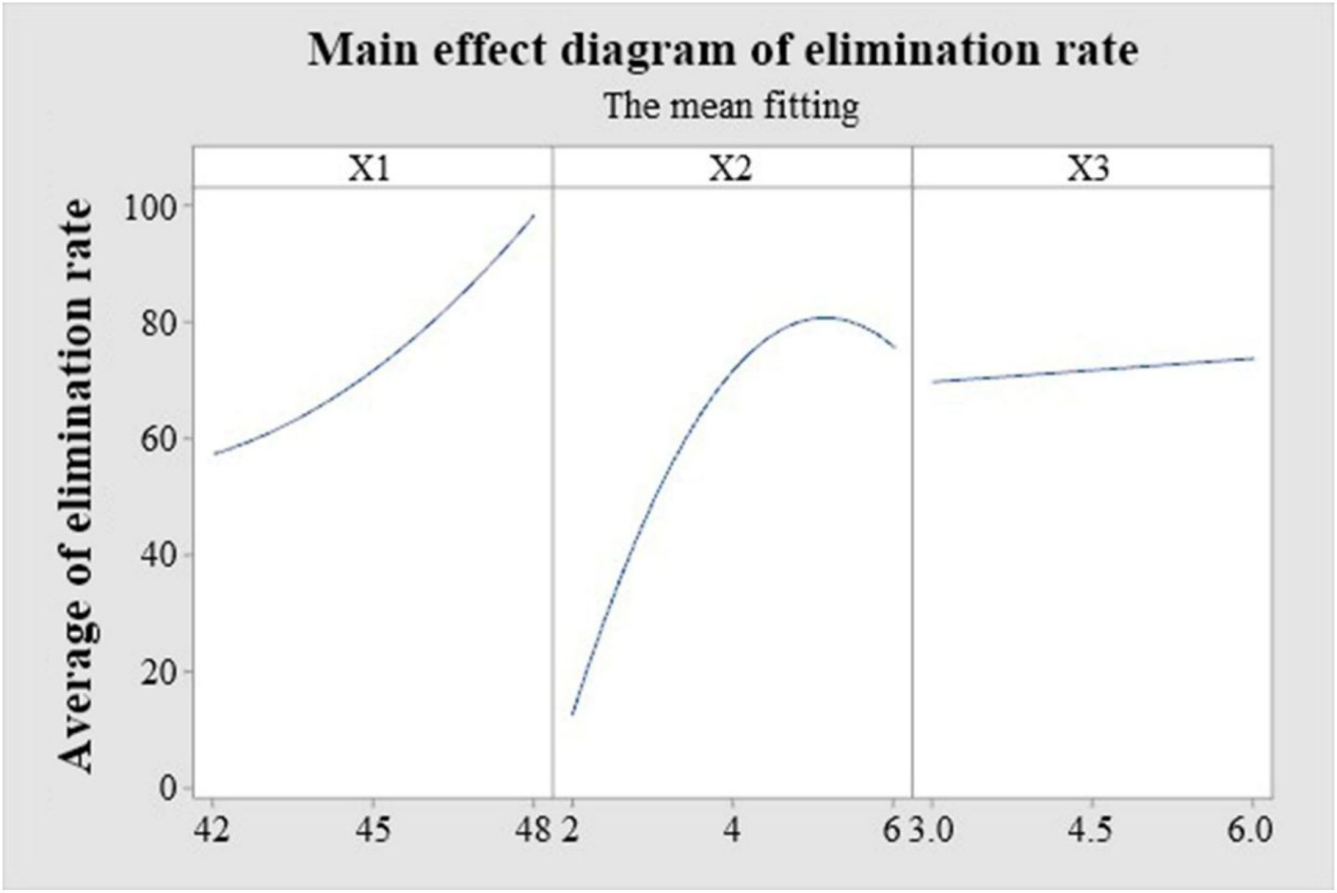
Uniform-design analysis results. X_1_ represented high-temperature; X_2_ represented high-temperature treatment time; X_3_ represented treatment time at 38 °C.

Quadratic regression of elimination rate on the three factors—upper temperature (X_1_), high-temperature pulse duration (X_2_), and 38 °C recovery duration (X_3_)—showed excellent fit (*R²* = 0.9978; adjusted *R²* = 0.9969; predicted *R²* = 0.9950; model p < 0.001). X_1_, X_2_, X_3_ and the quadratic terms of X_1_ and X_2_ were all significant (**Table 6**). Response profiles indicated that elimination increases with higher X_1_, is concave with X_2_ (rising to a maximum and then declining, with a downturn beyond ~5 h), and increases with longer X_3_ (**Figures 5**, **6**). The model accurately predicted near-complete elimination for D6-48-4–6 and D3-48-6-3 (both observed at 100%), but their vigor diverged: D6-48-4-6, which paired a shorter pulse at 48 °C with a longer 38 °C recovery, produced healthier shoot tips than D3-48-6–3 at the same elimination ceiling—highlighting the practical trade-off between eradication and plant quality. Guided by this trade-off, model-based optimization targeted > 99% elimination with minimal vigor loss, yielding a predicted optimum of 47 °C/5 h (dark) alternating with 38 °C/7 h (light).

**Table 6:** Variance and regression^*^ analysis of SCMV elimination rate.

Source	Degrees of freedom	Adj SS	Adj MS	*F* value	*P* value
Regression	5	28,647.1	5,729.42	1,080.91	0.000
X_1_	1	85.4	85.41	16.11	0.002
X_2_	1	3,704.2	3,704.17	698.83	0.000
X_3_	1	72.4	72.40	13.66	0.003
X_1_*X_1_	1	108.6	108.60	20.49	0.001
X_2_*X_2_	1	2,285.3	2,285.28	431.14	0.000
Error	12	63.6	5.30		
Total	17	28,710.7			
*R^2^*		0.9978			
Adjusted *R^2^*		0.9969	Predicted *R^2^*		0.9950

^*^Y= 940-53.4 ×X_1_ +71.01 ×X_2_ +1.337 ×X_3_ + 0.669 ×X_1_×X_1_-6.900×X_2_×X_2_.

### 3.5 Verification of the model-derived optimum

**Figure 7:**
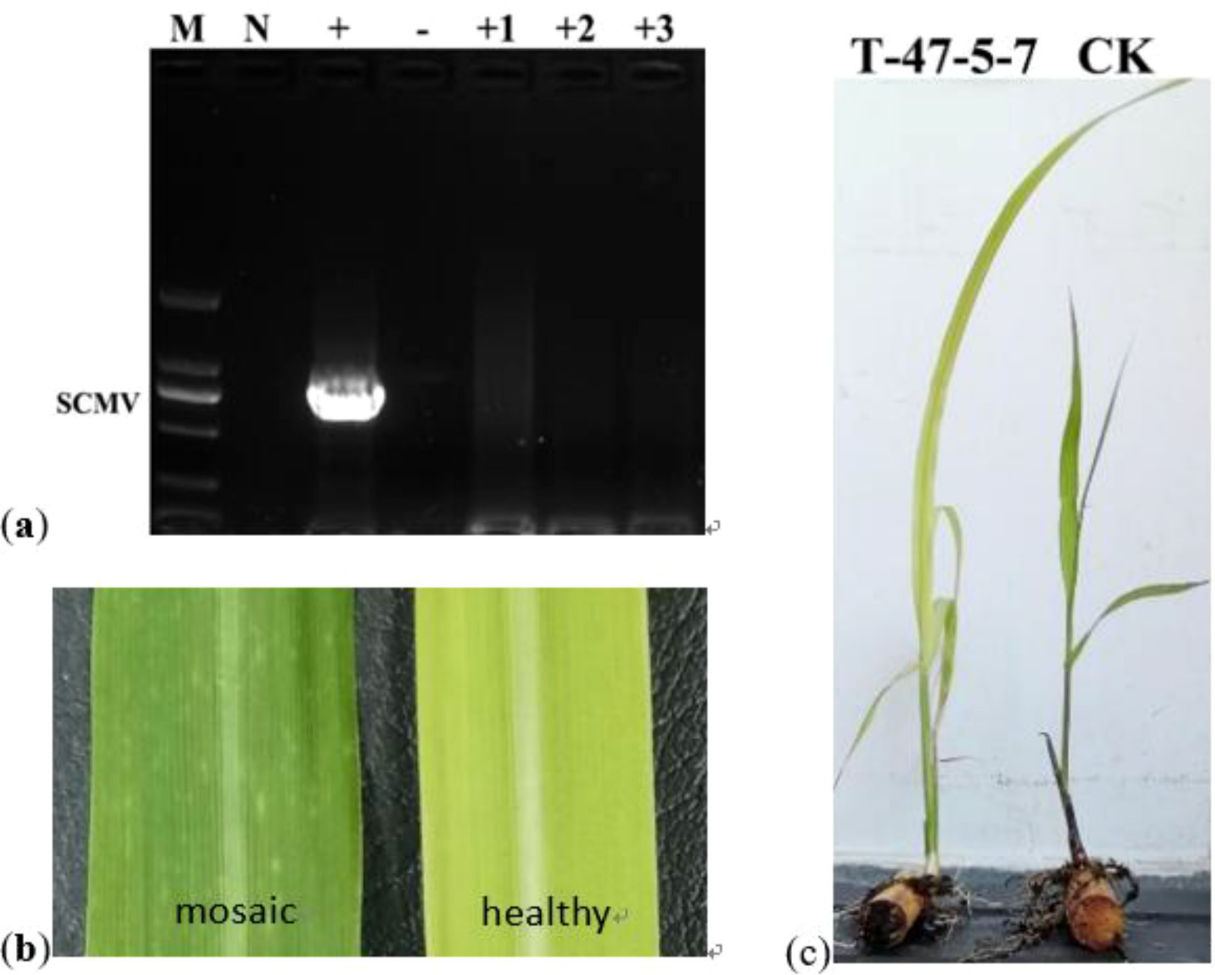
Verification of SCMV infection and symptom expression. **(a)** RT-PCR detection of SCMV in leaves collected from different positions. Lane M, 2000-bp DNA marker; Lane N, no-template control; Lane +, positive control (SCMV); Lane −, negative control; Lanes +1, +2, and +3, samples from the +1-, +2-, and +3-position functional leaves, respectively. **(b)** Visual comparison of leaf phenotypes: a leaf exhibiting mosaic symptoms typical of SCMV infection (left) versus a heavy leaf with uniform green coloration (right). **(c)** Phenotypic comparison of plants: AHTT-treated, SCMV-free plant (left; T-47-5-7) and SCMV-infected control plant (right; CK).

Because the optimum lay slightly beyond the tested X_3_ range (3–6 h), we treated the 7-h recovery as a model-guided extrapolation and validated it experimentally in independent replicates. The optimized AHTT (47 °C 5 h ↔ 38 °C 7 h, 12 days) was verified in triplicate. Sprouting percentage reached 97%, health index averaged 2, and RT-PCR of +1, +2, and +3 leaves was uniformly negative, confirming 100% elimination while maintaining acceptable vigor (**Figure 7**). Axillary-bud shoots were taller and stronger than the control and exhibited no mosaic symptoms, though transient leaf yellowing was observed during recovery—consistent with the physiological readouts described above.

## 4 Discussion

### 4.1 Physiological responses and oxidative stress under alternating high-temperature regimes

High-temperature stress disrupts membrane lipid homeostasis and triggers a sharp imbalance between the production and scavenging of reactive oxygen species (ROS), leading to cellular injury and metabolic dysfunction. Recent studies have revealed that membrane lipid reprogramming serves as a primary heat-sensing and signaling mechanism in plants such as wheat and rice, determining downstream thermotolerance through lipid desaturation and phospholipid remodeling (Hu et al., 2023; Sharma et al., 2023). Under heat stress, the phase transition of thylakoid and plasma membranes enhances permeability, thereby facilitating oxidative burst and impairing photosynthetic electron transport.

In this study, *S. officinarum* cv. Xuezhe axillary-bud shoots exhibited a pronounced rise in catalase (CAT) activity and a sharp reduction in peroxidase (POD) activity as thermal intensity increased, consistent with reports that CAT is more heat-stable and inducible under severe stress, whereas POD is thermolabile and its overactivity may exacerbate oxidative injury (Détain et al., 2022; Fortunato et al., 2023). CAT efficiently detoxifies excess H_2_O_2_ without producing additional free radicals, while POD-mediated ROS removal can itself yield phenoxy radicals that intensify peroxidative damage. The contrasting responses of these two enzymes therefore reflect a self-protective metabolic reconfiguration: plants under AHTT favor CAT-dependent detoxification to maintain redox equilibrium and minimize cellular injury.

Importantly, this physiological trade-off highlights why a “one-size-fits-all” constant high-temperature regime may be suboptimal for heat-sensitive germplasm. In such cultivars, temperature scheduling that incorporates recovery phases may result in a more favorable redox balance and higher propagule survival, thereby enabling effective sanitation without unacceptable physiological costs.

### 4.2 Chloroplast ultrastructure and chlorophyll degradation

Chloroplasts are among the most heat-sensitive organelles. Elevated temperatures disturb chlorophyll biosynthesis, destabilize thylakoid membranes, and accelerate pigment photobleaching. Recent cryo-electron microscopy studies have shown that heat-induced thylakoid unstacking and grana disassembly lead to functional collapse of the photosystem II reaction center (Kirchhoff, 2014; Yamamoto, 2016; Mazur et al., 2021; Gu et al., 2022). The marked decline in nitrogen balance index (NBI) and chlorophyll content observed here supports this mechanistic interpretation. As AHTT duration exceeded 4–6 h, chlorophyll synthesis was suppressed and leaf chlorosis intensified, paralleling findings in *Oryza sativa* and *Sesamum indicum* where sustained exposure to ≥45 °C caused irreversible pigment degradation and photoinhibition (Su et al., 2022; Chandarak et al., 2023; Rong et al., 2024).

These structural and physiological responses are not merely passive damage but may act as controlled downregulation of photosynthetic metabolism to reduce oxidative load. Transcriptomic analyses of heat-stressed sugarcane have shown that chlorophyll a/b binding proteins, Rubisco activase, and electron-transport components are transiently suppressed during thermotherapy to prevent excess ROS accumulation (Yang et al., 2022; Barratt et al., 2024). Thus, the reduced chlorophyll and NBI under AHTT likely reflect an adaptive rebalancing between energy capture and stress mitigation rather than irreversible injury when exposure is optimally controlled.

### 4.3 Mechanistic insights into SCMV elimination by AHTT

Thermotherapy is known to suppress viral replication by disturbing replication complexes and inhibiting intercellular movement. Potyviruses such as SCMV rely on endoplasmic-reticulum (ER)-derived vesicles for replication and plasmodesmata (PD) for cell-to-cell movement via virus-encoded movement proteins (MPs). Temperature can selectively impair viral cell-to-cell movement. For example, *Tobacco mosaic virus* mutants carrying single-amino-acid substitutions in the movement protein lose mobility at approximately 33 °C while retaining replication competence (Liu and Nelson, 2013), and ALSV is eliminated after prolonged culture at 37 °C (Yamagishi et al., 2016). Our finding that complete SCMV elimination occurred at alternating regimes above 47 °C strongly suggests a comparable temperature-dependent functional deactivation of SCMV MP or associated replication factors.

Moreover, high-temperature exposure may activate host antiviral RNA-silencing pathways. Thermotherapy has been shown to upregulate *DCL2/4*, *AGO1*, and *RDR6* genes and enhance the accumulation of virus-derived small interfering RNAs (vsiRNAs) in *Pyrus pyrifolia* and *Solanum lycopersicum* (Ammara et al., 2015; Liu et al., 2016). The AHTT conditions likely intensified this antiviral response, resulting in targeted degradation of viral RNAs and cessation of systemic spread. Recent omics-based studies propose that heat-induced RNA silencing and membrane disruption act synergistically to eradicate viruses while allowing recovery of meristematic tissues (Bettoni et al., 2022b; Glushkevich et al., 2022).

### 4.4 Beyond SCMV: multi-virus considerations

We acknowledge that sugarcane can harbor multiple viruses (e.g., SrMV and SCSMV) and that mixed infections are common in field-grown materials. In this study, we focused on SCMV because it was the predominant sanitation target in our system and the primary virus associated with mosaic symptoms in cv. Xuezhe; therefore, the performance of the optimized AHTT regime against other viruses or mixed infections cannot be concluded from the present data. Nevertheless, the model-guided AHTT approach is not conceptually restricted to SCMV: because thermotherapy can disrupt viral replication and movement and can enhance host antiviral defenses, the same uniform-design and response-surface framework can be extended to other virus–host combinations, provided that virus-specific or multiplex diagnostics are incorporated as endpoints. Future validation using multiplex RT-PCR for SCMV, SrMV, and SCSMV (including mixed infections) will be essential to determine whether a single alternating regime offers broad-spectrum sanitation or whether virus- and cultivar-specific optimization is required.

### 4.5 Integrative implications of alternating temperature thermotherapy and broader applicability

Building on these mechanistic insights and considering practical multi-virus contexts, compared with constant-temperature heat treatment, the alternating high-temperature (AHTT) system combines viral inactivation with intermittent recovery phases at 38 °C, permitting cellular repair and enhancing survival. Alternating regimes are known to maintain higher tissue viability by allowing protein refolding, ROS scavenging, and lipid remodeling during the lower-temperature phase (Zhao et al., 2018; Karimpour et al., 2020). Our uniform-design analysis captured strong non-linear effects of temperature scheduling, indicating that a short high-temperature pulse combined with a longer recovery phase can achieve complete SCMV elimination while preserving vigor. This supports the concept that oscillatory thermoregulation can be biologically advantageous for sanitation when heat injury is a primary constraint.

The regression model revealed pronounced non-linear effects of temperature and pulse duration, consistent with observations from thermotherapy–cryotherapy hybrid systems in which viral clearance often follows a sigmoidal dose–response relationship (Wang et al., 2008; Zhao et al., 2018). Accordingly, the optimized AHTT regime (47 °C × 5 h dark/38 °C × 7 h light for 12 days) achieved complete SCMV removal with minimal physiological cost, offering a practical balance between virus inactivation and host survivability. From an application standpoint, the added value of this model-guided AHTT strategy is expected to depend on cultivar heat sensitivity: for heat-tolerant cultivars, conventional constant high-temperature thermotherapy (e.g., ≥52 °C) may be sufficient and simpler to implement, whereas for heat-sensitive cultivars such as Xuezhe, a data-driven optimization approach offers a rational route to identify alternating regimes that remain antiviral while minimizing heat injury. Thus, although our experimental validation was conducted in cv. Xuezhe, we consider the principal contribution to be a transferable optimization framework applicable to other heat-sensitive, vegetatively propagated materials where the trade-off between virus elimination and thermal damage is critical; nevertheless, extension to additional cultivars and virus backgrounds will require empirical validation.

### 4.6 Future perspectives and applications

The successful application of AHTT against SCMV provides a methodological basis for refining thermotherapy protocols in vegetatively propagated systems, particularly where heat sensitivity limits the feasibility of constant high-temperature treatment. Future work should prioritize validation across additional sugarcane cultivars spanning a range of thermotolerance, as well as mixed-virus contexts (e.g., SrMV and SCSMV), ideally using multiplex diagnostics. Where rapid multiplication is required after sanitation, AHTT can be integrated with optional downstream regeneration approaches (e.g., meristem-tip culture or somatic embryogenesis), as demonstrated in apple, pear, and potato systems (Hu et al., 2012; Bettoni et al., 2022b). High-resolution imaging and multi-omics profiling could further elucidate chloroplast remodeling, plasmodesmatal function, and RNA-silencing activation under AHTT, helping to refine regime design for different host–virus combinations.

In the context of rising global temperatures, elucidating plant–virus–heat interactions is increasingly important (Hsieh and Chiou, 2013; Chiou et al., 2015; Hsieh, 2016; Hsieh et al., 2023). Heat-triggered antiviral defenses involving ROS signaling, membrane fluidity modulation, and RNA silencing represent promising targets for both sanitation and crop improvement. Overall, our results illustrate how controlled abiotic stress can be leveraged as a practical, scalable, and eco-friendly tool for managing virus disease in heat-sensitive vegetatively propagated crops.

## 5 Conclusion

This study establishes an optimized alternating high-temperature thermotherapy (AHTT) protocol that completely eliminates Sugarcane mosaic virus (SCMV) from the chewing-cane cultivar *S. officinarum* cv. Xuezhe while maintaining strong plantlet vigor. Using a uniform-design framework and quadratic regression modeling, we quantified the effects of temperature, pulse duration, and recovery phase on both virus elimination and plant physiology.

The optimal AHTT regime—47 °C for 5 h (dark) alternating with 38 °C for 7 h (light) for 12 days—produced 100% virus-free shoot tips from rooted cuttings with 97% sprouting percentage and an average health index of 2. Physiological assays revealed a heat-induced redox reprogramming characterized by increased catalase (CAT) and reduced peroxidase (POD) activity, mitigating oxidative stress and maintaining tissue viability.

The success of AHTT likely results from a synergistic combination of replication-complex disruption, movement-protein inactivation, and enhancement of RNA silencing, supported by recovery-phase repair of membranes and chloroplasts. This alternating regimen thus reconciles viral lethality with host resilience—an advantage over conventional constant-temperature approaches.

Overall, AHTT represents a next-generation thermotherapy paradigm that integrates physiological understanding with statistical optimization to achieve predictable, reproducible, and eco-friendly viral eradication. Its scalability makes it suitable for mass propagation of virus-free sugarcane and adaptable to other vegetatively propagated crops. Future work should combine AHTT with meristem-tip culture or cryotherapy and employ multi-omics analyses to elucidate molecular mechanisms of thermotolerance and viral clearance, providing a foundation for climate-resilient and pathogen-free agriculture.

## Data Availability

The raw data supporting the conclusions of this article will be made available by the authors, without undue reservation.
